# The outcome and cost-effectiveness of nurse-led care in the community for people with rheumatoid arthritis: a non-randomised pragmatic study

**DOI:** 10.1136/bmjopen-2015-007696

**Published:** 2015-08-25

**Authors:** Richard A Watts, Janice Mooney, Garry Barton, Alex J MacGregor, Lee Shepstone, Lisa Irvine, David G I Scott

**Affiliations:** 1 Norwich Medical School, University of East Anglia, Norwich, UK; 2 School of Nursing Sciences University of East Anglia, Norwich, UK

**Keywords:** RHEUMATOLOGY, HEALTH ECONOMICS

## Abstract

**Objective:**

To determine the outcome and cost-effectiveness of nurse-led care in the community for people with rheumatoid arthritis (RA).

**Design:**

Non-randomised pragmatic study.

**Setting:**

Primary (7 primary care practices) and secondary care (single centre) in the UK.

**Methods:**

In a single area, pragmatic non-randomised study, we assessed the outcome, cost-effectiveness of community-based nurse-led care (NLC) compared with rheumatologist-led outpatient care (RLC). Participants were 349 adults (70% female) with stable RA assessed at baseline, 6 and 12 months. In the community NLC arm there were 192 participants. Outcome was assessed using Stanford Health Assessment Questionnaire (HAQ). The economic evaluation (healthcare perspective) estimated cost relative to change in HAQ and quality-adjusted life years (QALY) derived from EQ-5D-3L. We report complete case and multiple imputation results from regression analyses.

**Results:**

The demographics and baseline characteristics of patients in the community group were comparable to those under hospital care apart from use of biological disease-modifying antirheumatic drugs (DMARDS), which were adjusted for in the analysis. The mean incremental cost was estimated to be £224 less for RLC compared to the community NLC, with wide CIs (CI –£213 to £701, p=0.296). Levels of functional disability were not clinically significantly higher in the community NLC group: HAQ 0.096 (95% CI −0.026 to 0.206; p=0.169) and QALY 0.023 (95% CI −0.059 to 0.012; p=0.194).

**Conclusions:**

The results suggest that community care may be associated with non-significant higher costs with no significant differences in clinical outcomes, and this suggests a low probability that it is cost-effective.

**Strengths and limitations of this study**

- Observational study of the cost-effectiveness and outcomes of community nurse-led care for rheumatoid arthritis, thereby reflecting real life care.
- Include 349 patients followed for 12 months.
- Compliments studies of nurse-based hospital care.
- Single area which may limit generalisability.
- Final recruitment less than planned.

## Introduction

Rheumatoid arthritis (RA) is a common chronic destructive arthropathy. The overall prevalence of RA in the UK is 0.81%,^1^ with the majority of patients requiring long-term treatment with immunosuppressant drugs. In 2009, it was estimated by the national audit office that RA cost the National Health Service (NHS) £560 million per year.^2^

The traditional model of RA treatment is for all follow-up to be conducted by rheumatologists in hospital departments; however, NHS policy currently favours moving chronic disease management from hospital-based clinics into the community.^3^ Over the past three decades, rheumatology nurse practitioners (RP) (also known as clinical nurse specialists) have become an accepted part of the multidisciplinary team and complete many tasks traditionally performed by rheumatologists, including joint injections and prescribing.^4^ ^5^ RPs assess disease activity, monitor effects of therapy, provide patient education, psychological support, care coordination and some RPs can prescribe medication and recommend medication changes.^6^ ^7^ Patients attending clinics with nurse led care (NLC) have good outcomes in terms of physical function, disease activity, quality of life, pain, fatigue, stiffness, psychological function and satisfaction.^8–11^ Convenience, continuity of care, and proximity of services to home are considered advantages of community NLC.^12^ However, patients with RA need ready access to other members of the multidisciplinary team such as physiotherapists and occupational therapists. Thus, it is not clear whether the move to community care will disrupt the use and development of multidisciplinary teams.

Various models of multidisciplinary care were investigated in a multicentre study in Holland and NLC was shown to be more cost-effective than inpatient care or day patient care.^13^ The cost-effectiveness and outcome of nurse-led care in the UK has been evaluated in a multicentre randomised controlled trial in a secondary care setting and shown to be not inferior to rheumatologist-led care (RLC).^14^ A recent study from Denmark suggested that shared care and nurse-led care cost less and provided broadly similar health outcomes compared with rheumatologist outpatient care.^15^ For consultants working in community-based clinics, it seems that increased health benefits are achieved at increased cost;^16–18^ the same may not be true of nurses whose employment costs are substantially lower than doctors. What is not known is the cost-effectiveness of community NLC, compared to traditional RLC.

In Norfolk, services have evolved with the development of community NLC based in primary care, which are entirely nurse led, without direct support from a consultant. Thus, the opportunity arose to address the research question as to the cost-effectiveness of community NLC compared with RLC by means of a pragmatic observational study.

## Methods

The aim of this study was to evaluate the health outcomes and costs of RA services provided in primary and secondary care. We designed an observational non-randomised study to evaluate the clinical and cost-effectiveness of independent community NLC, in comparison with standard RLC. We did not wish to disrupt or alter the pattern of care and therefore, choose an observational non-randomised study design.

### Study population

Patients with established RA undergoing routine follow-up. Patients fulfilled the American College of Rheumatology (ACR) criteria for RA^19^ and were aged over 18 years. We excluded patients with new onset RA (less than 1-year duration) or with severe functional impairment or very active unstable disease as such patients would tend to be solely cared for in secondary care. Patients were recruited at the time of their routine secondary care outpatient or community clinic appointment. We recruited from all the clinics in primary care and from the main secondary care centre to minimise selection bias.

#### Structure of rheumatology services in Norfolk

Norfolk is a rural county in the East of England with a central secondary care hospital and satellite community hospitals in addition to primary care centres. Patients often have to travel significant distances to access secondary care and hence, community-based care is more convenient for them.

#### Community clinics

Nurse-led care in the community was provided by five RPs in seven general practices (6 rural and 1 urban within Norwich), with clinics held every 1–4 weeks. These were nurse-led and independent of direct rheumatologist input. Patients seen in these clinics were only referred for a consultant opinion when needed. The RPs assessed patients’ disease activity and their drug therapy for efficacy and side effects; however, prescriptions were provided by their general practitioners, and patients requiring joint injections could access these via the main secondary care hospital or the two community hospitals.

#### Hospital clinics

The secondary care service was provided by five consultant rheumatologists, supported by specialist registrars and RPs, based in one large teaching hospital and two community hospitals. The RPs worked alongside the medical staff but did not provide independent NLC. For most of the study there were five RPs, some of whom also worked in the primary care clinics. There was also access to a day unit for intravenous therapies and intra-articular injections, together with a full multidisciplinary team.

#### Sample size calculation

Based on a small difference between the groups (a Cohen's Effect Size of 0.35), 175 participants in each group were required to confer 90% power without adjustment for potential confounders. With an adjustment (using a general linear model) for a potential confounding imbalance in HAQ at baseline and assuming that the coefficient of determination between groups was no more than 0.25, then 235 participants per group were required to confer 90% power.

### Estimatation of costs

#### Measuring resource use

We used a modified version of a previously developed resource-use questionnaire^20^ administered at baseline, 6 months and 12 months, participants were asked to report the following items of resource use over the previous 6-month period:

- contacts with health professionals
- arthritis-related admissions to hospital or day-units
- arthritis-related tests and procedures, including steroid injections
- arthritis-related prescribed medications—specifically biological disease-modifying antirheumatic drugs (DMARDs), non-biological DMARDs and opioid analgesics
- time taken off work due to their arthritis
- travel costs associated to contact with healthcare professionals

#### Assigning costs to items of resource use

Costs were estimated using 2011/2012 financial year levels; NHS and personal social services (PSS) costs were taken from a combination of Curtis^21^ and the National Schedule of Reference Costs,^22^ (see online supplementary table S1). Participant travel costs were estimated using a cost of £0.40 per mile for private car; specific fees were requested for those who used public transport or a taxi. Where full details of the usual method of travel were not provided, mean imputation was used, to estimate an overall travel cost for each participant.

The average rate of hourly earnings (in 2011)^23^ was used to estimate the cost associated with lost productivity.^24^ However, since productivity costs may vary if different costing methodologies were applied,^25^ these costs are reported separately to the other aforementioned costs, in subsequent sensitivity analysis.

Participants were asked to report the name, dose and frequency of prescribed medications, and the number of prescriptions in the past 6 months. We assigned costs to those considered taken for RA purposes. Non-steroidal anti-inflammatory drugs (NSAIDs) were excluded as we could not discriminate between those used for RA or other purposes. Unit costs were taken from the prescription cost analysis.^26^

### Overall costs

Total healthcare costs, for both the community and hospital groups, were estimated by summing the costs associated with the aforementioned items of resource use. Overall costs were estimated by summing total healthcare, travel and lost productivity costs.

### Measuring outcomes

We used the Stanford Health Assessment Questionnaire (HAQ) to assess disability. We report cost-effectiveness in terms of the incremental cost of community delivery per incremental change on the HAQ scale. Participants completed the HAQ at 0, 6 months and 12 months.

To estimate levels of health-related quality of life, participants completed the EQ-5D-3L^27^ at baseline, 6 months and 12 months. The York A1 tariff was applied to these responses in order to enable an associated utility score (this has a scale where 0=death and 1=full health) to be calculated.^28^ The area under the curve method^29^ was used to estimate the total quality adjusted life year (QALY) score over the 12-month follow-up period, where it was assumed EQ-5D scores changed linearly between time points. Completion of the EQ-5D enabled a cost-utility analysis to be undertaken using cost per QALY gained.

### Economic analysis

Costs were calculated from the healthcare perspective (NHS and Personal Social Services)^30^ and included only those participants who returned both the 6-month, and 12-month follow-up questionnaire. We compared the cost-effectiveness of nurse-led clinics in the community (‘community’) to hospital-based outpatient care (‘hospital’). As all trial follow-up was completed within 12 months, no discounting was applied to either costs or outcomes.

In the primary analysis, a complete case approach was adopted, where complete cost and outcome data (either HAQ/QALYs based on the EQ-5D) was required. However, in order to include all available data, multiple imputation was undertaken as part of a sensitivity analysis. We imputed missing (disaggregated) costs, EQ-5D and HAQ data at each time point, following *mi* procedures in StataSE 12. Imputation took place in five cycles, the estimates from which were then pooled and calculated using Rubin's rules.^31^ ^32^

Bivariate linear regression was used to estimate the mean cost difference (incremental cost) between the two groups (this was estimated for total healthcare and overall costs), and the mean incremental effect (difference in QALYs/HAQ), where the baseline costs, outcome measures (EQ-5D-3L/HAQ), age and gender acted as covariates.^33^ In order to estimate the level of uncertainty associated with the decision regarding cost-effectiveness, we also estimated 95% CIs and the cost-effectiveness acceptability curve (CEAC). Using a bootstrapped sample, the probability that the community arm had a higher net benefit compared to hospital care (vertical axis) was plotted for each value of λ (horizontal axis). We also graphically represent the bootstrapped cost-effect pairs on a cost-effectiveness plane.^34–37^

## Results

### Participants

**Figure 1:**
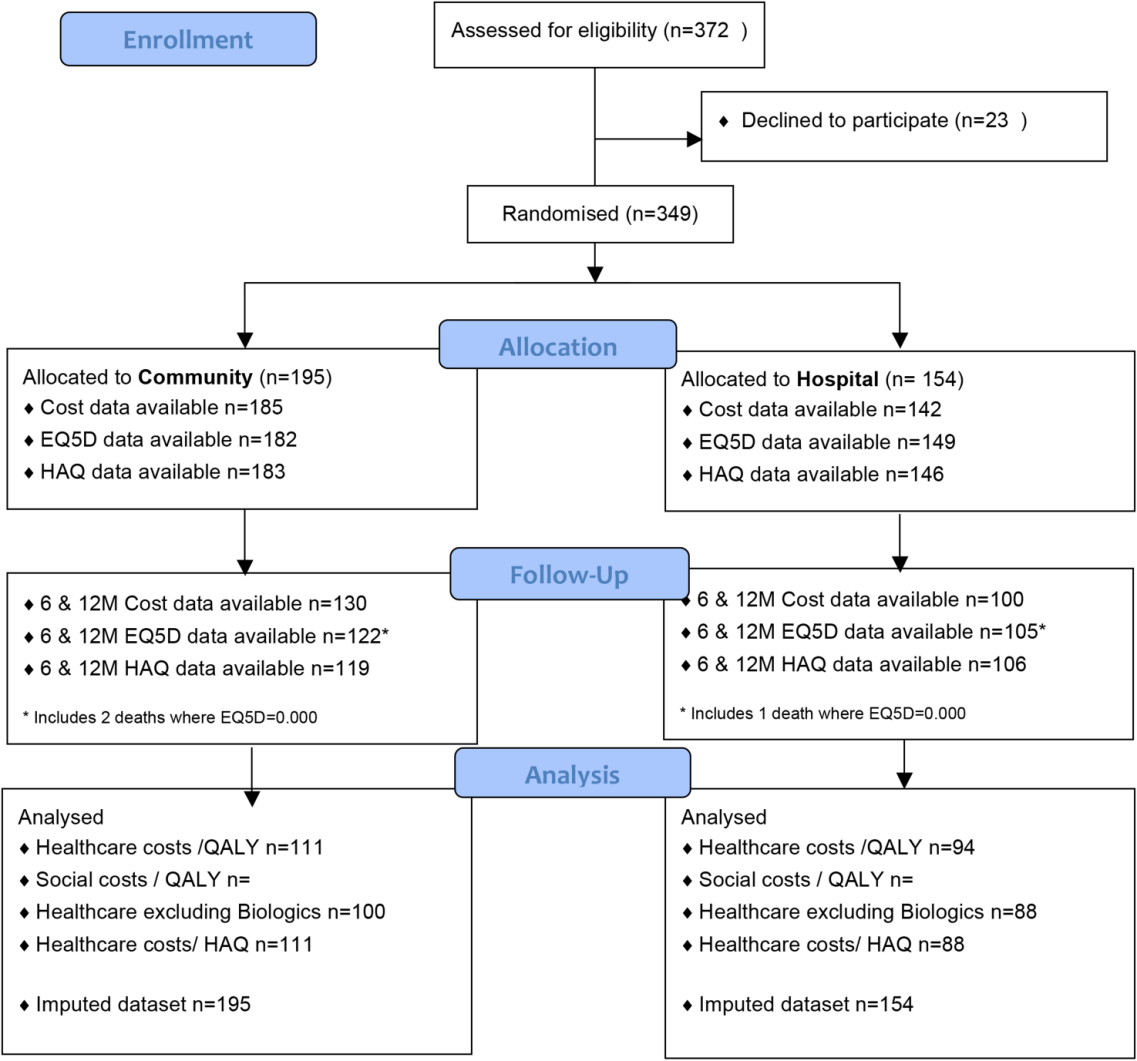
Patient flow through the study.

We approached 204 patients in the community and 168 in the hospital arm. Primary analysis was on complete cost and QALY data at all three time points, which consisted of 205 participants (111 in community arm; 94 in hospital arm). Participant flow through the study is shown in figure 1, and basic demographic data in table 1.

**Table 1:** Baseline characteristics of study population stratified by study group

Baseline characteristics	Community (n=192)	Hospital (n=154)
Women, n (%)	136 (70.10)	106 (69.28)
Age, years; mean (SD)	65.56 (10.56)	64.86 (11.39)
Disease duration, years; mean (SD) (n=172; 147)	13.82 (11.33)	12.91 (11.45)
Baseline RA regimen		
Methotrexate (%)	155 (80.7)	128 (83.1)
Sulfasalazine (%)	38 (19.8)	25 (16.2)
Hydroxychloroquine (%)	40 (20.8)	40 (26.0)
Leflunomide (%)	17 (8.8)	12 (7.8)
Prednisolone (%)	25 (13.0)	22 14.3)
Biological DMARDs (%)	17 (8.8)	3 (1.9)
Opioid analgesics (%)	43 (22.4)	31 (20.1)
Baseline outcome scores, mean (SD)		
HAQ (182; 149)*	1.01 (0.75)	0.98 (0.78)
EQ-5D-3L (183; 146)*	0.635 (0.258)	0.624 (0.307)

*Figures in brackets refer to the number of completed forms for each group.

DMARDs, disease-modifying antirheumatic drugs; HAQ, Health Assessment Questionnaire; RA, rheumatoid arthritis.

### Costs

For those who completed the cost questionnaires, mean level of health resource use and costs for each of the two groups are summarised in table 2, with further details on health professional visits summarised in online supplementary table S2. Mean health professional contact costs were marginally higher for the hospital group (£642 compared with £581). There was little difference between the two groups with regard to the mean cost associated with hospital/day-case admissions, steroid use or tests and procedures.

**Table 2:** Mean (range) levels of resource use and associated costs

	Levels of resource use		Mean cost (£)	
Item	Community n=130	Hospital n=100	Community	Hospital
Health professional contacts	14.6 (1 to 69)	16.7 (1 to 63)	£581 (£41 to £5574)	£642 (£40 to £3917)
Hospital/day-case admissions*	0.14 (0 to 5)	0.18 (0 to 11)	£213 (£0 to £6435)	£209 (£0 to £5958)
Tests and procedures*	2.7 (0 to 10)	2.6 (0 to 9)	£33 (£0 to £230)	£34 (£0 to £196)
Steroid injections	0.57 (0 to 7)	0.45 (0 to 4)	£73 (£0 to £875)	£44 (£0 to £500)
NHS costs excluding medications	–	–	£900 (£53 to £7655)	£928 (£44 to £7678)
Biological DMARDs	0.13	0.04	£1210 (£0 to £9295)	£323 (£0 to £9295)
Non-biological DMARDs	1.41	1.43	£330 (£0 to £3063)	£332 (£0 to £1554)
Opioid analgesics	0.16	0.18	£27 (£0 to £745)	£21 (£0 to £577)
Overall NHS costs			£2467 (£58 to £16 811)	£1604 (£153 to £13 835)
Travel			£62 (£0 to £840)	£69 (£0 to £488)
Time off work (days)*	3.26 (0 to 160)	3.71 (0 to 182)	£306 (£0 to £15 698)	£329 (£0 to £17 856)
Overall NHS and lost productivity costs	–	–	£2835 (£61 to £16 953)	£1975 (£153 to £18 806)

*Based on complete case data set.

DMARDs, disease-modifying antirheumatic drugs; NHS, National Health Service.

Travel costs were marginally lower in the community arm compared with hospital (£62 vs £69). In the community group, 93.5% reported taking no time off work (due to their arthritis) compared with 85.8% of those in the hospital group in the 12-month follow-up period, the mean values equated to 3.26 days and 3.71 days, respectively.

Overall, health resource use associated with community NLC was very similar to that in hospital-based care apart from one aspect: prescribed medications. At baseline, RA participants in community arm were using £553 medications over 6 months, compared to £223 in hospital. Over the 1-year follow-up, this disparity had increased to £1567 per participant in the community arm compared with £676 for hospital-treated patients. The largest cost difference appeared with biological DMARDS, but for opioid analgesics, costs were also slightly higher in the community arm compared with hospital- based participants.

### Outcomes

#### EQ-5D-3L

EQ-5D scores at baseline, 6 and 12 month are summarised in table 3. Hospital participants EQ-5D outcomes improve slightly (+0.043) over the 12-month follow-up, whereas community participants EQ-5D outcomes decline slightly (−0.015) over same period. This represents an incremental QALY gain of 0.026 QALYs in the hospital arm compared to community, which is negligible in terms of quality of life impact.

**Table 3:** Estimates of the mean EQ-5D and HAQ over the 12-month follow-up period

	EQ-5D and QALY score		HAQ score	
	Community	Hospital	Community	Hospital
	n=122	N=105	N=119	N=106
Baseline	0.654 (–0.181 to 1)	0.646 (–0.184 to 1)	0.956 (0 to 3)	0.913 (0 to 3)
6 month	0.633 (–0.073 to 1)	0.646 (–0.181 to 1)	1.058 (0 to 2.88)	0.925 (0 to 3)
12 month	0.639 (–0.181 to 1)	0.689 (–0.015 to 1)	1.061 (0 to 2.88)	0.921 (0 to 3)
QALY score	0.639 (–0.071 to 1)	0.665 (–0.086 to 1)		

HAQ, Health Assessment Questionnaire; QALY, quality-adjusted life years.

### Health Assessment Questionnaire

HAQ outcomes were compared for participants who completed both baseline and 12-month assessments (119 in community arm, 106 in hospital; table 3). At baseline, the mean HAQ score was slightly higher in the community arm (0.956 compared with 0.913 in hospital). At 12 months, disability had progressed further in the community arm (1.061 compared with 0.921 in hospital). Based on the regression analysis, it was estimated that there was a 0.096 difference in progression (scores had worsened more in the community arm compared to the hospital). This is well below the minimal clinical important difference of 0.22.^38^

### Cost-effectiveness analysis

Based on those with complete cost and EQ-5D data, the mean incremental total NHS cost was estimated to be £224 less for the hospital group compared to the community group (CI –£213 to £701, p=0.296; table 4). When travel costs and lost productivity (time off work) costs were included, the incremental cost was £917 (CI £145 to £1688, p=0.020). The incremental QALY gain (for the community arm compared to the hospital) was negligible, estimated to be −0.023 (–0.059 to 0.012, p=0.194). This demonstrates no significant difference between each of the groups with regard to either healthcare costs, travel, lost productivity or overall costs or QALYs. When comparing the healthcare costs to clinical outcomes, as measured by the HAQ, we found the community group had higher mean costs (mean difference=£127, CI −£369 to £624, p=0.589) and a higher level of functional disability (mean difference=0.096 CI −0.026 to 0.206, p=0.169). In all analyses, outcomes (QALYs or HAQ) were worse in the community arm; however, the difference was negligible. The community group had higher mean overall costs and lower mean effect. These findings were consistent when using the imputed (full) data set.

**Table 4:** Cost-effectiveness results

Complete case data set	Costs (community compared to Hospital)					Outcomes (community compared to Hospital)			
Cost utility(cost/QALY)	N	Costs	CI		p Value	QALY*	CI		p Value
NHS costs	199	£244.24	−213	701	0.296	−0.023	−0.059	0.012	0.194
NHS and social costs	199	£917.21	145	1688	0.022	−0.023	−0.056	0.016	0.194
Excludes patients on biological DMARDs	185	£234.40	−201	669	0.376	−0.026	−0.055	0.018	0.259
Healthcare excluding medication costs	199	£51.07	−324	426	0.790	−0.024	−0.058	0.010	0.167
NHS costs, adjusting for baseline biologics	199	£241.13	−217	699	0.302	−0.023	−0.056	0.016	0.194
NHS+social costs, adjusting for baseline biologics	199	£488.71	−219	1197	0.176	−0.023	−0.056	0.016	0.194

Cost-effectiveness (cost/HAQ)		Costs	CI			HAQ†	CI		

NHS costs	194	£127.09	−369	624	0.589	0.096	−0.026	0.206	0.169
NHS and social costs	194	£759.86	−38	1558	0.062	0.073	−0.050	0.195	0.248
Excludes patients on biological DMARDs	177	£240.76	−501	983	0.525	0.076	−0.054	0.206	0.251
Healthcare excluding medication costs	194	£73.72	−573	719	0.737	0.086	−0.035	0.207	0.165

Bivariate regression of cost variable on treatment arm, baseline costs, age and sex; outcome variable on treatment arm, baseline outcome, age and sex, with correlation. Tested against no difference between groups.

Lower QALY scores denote reduced quality of life in the community arm compared to hospital.

*Higher HAQ scores denote worse clinical outcomes in the community arm compared to hospital.

†Higher costs—community arm is more expensive compared to hospital.

### Sensitivity analysis

To account for the marked impact of biological DMARD costs to overall cost-effectiveness, we also report healthcare costs excluding medication costs, NHS costs adjusting for baseline biologics, and NHS and social costs adjusting for baseline biological use (table 4). Excluding those who received biologics at baseline, the community arm continued to cost more than hospital care, but at a lesser margin of £234 (CI −£201 to £669) less using cost/QALY, and £241 (CI −£501 to £983) less than hospital care using cost/HAQ.

### CE plane

**Figure 2:**
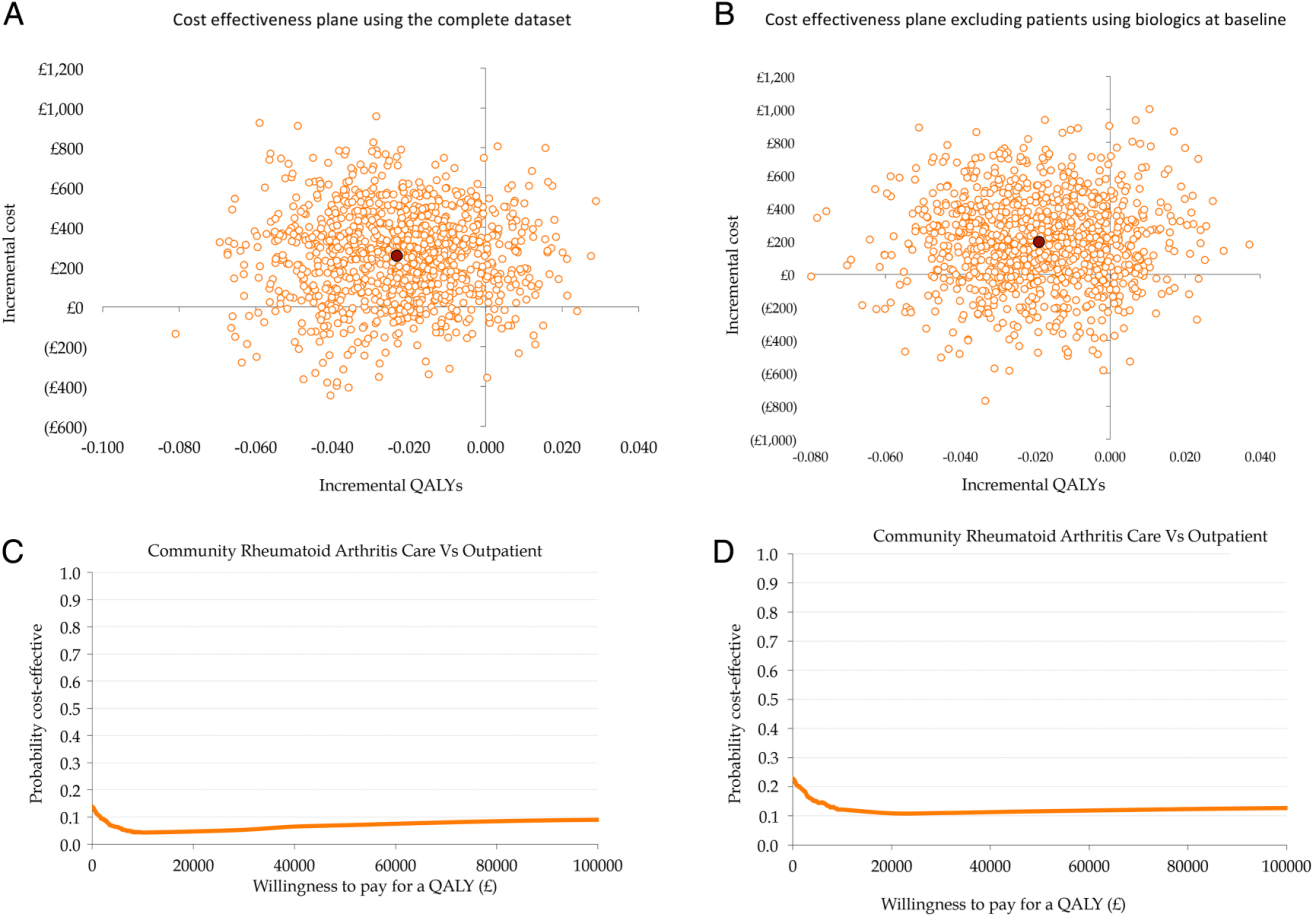
(A and B) Cost-effectiveness plane (complete data set); (C and D) Cost-effectiveness acceptability curve (complete data set).

The cost-effectiveness plane for 2000 bootstrap replications comparing cost and QALYs of community RA care compared to hospital setting is shown in figure 2 and B. Most cost-effect pairs are located in the north-west quadrant, suggesting community care is associated with higher costs and unfavourable outcomes. Data from the multiple imputed data set is provided in online supplementary figure S1A.

The cost-effectiveness acceptability curve (CEAC) is shown in figures 2C and D. With a probability of being more cost-effective than hospital care in less than 10% at all thresholds, community care is clearly less favourable. Data from the multiple imputed data set is provided in the online supplementary figure 1B.

## Discussion

This study is the first to provide data on the cost-effectiveness of routine community NLC of patients with RA. We compared patients with stable RA managed in a community NLC setting with those managed in traditional RLC. The two groups were reasonably well matched at baseline in terms of disease severity. Baseline imbalance with medication use was adjusted for in sensitivity analysis. We found that care in the community was associated with a non-significant higher mean cost and no clinically significant change in effectiveness, as assessed by EQ-5D and HAQ.

The number and subsequent cost of health professional visits and hospital admissions was very similar between study arms. There was no evidence in our study that access to physiotherapists, occupational therapists and podiatrists was disrupted by care in the community. Drug monitoring also was carried out to the same extent with no evidence that visits to phlebotomists was changed. The number of RP visits was greater in the community. Surprisingly, given the model of care, the number of rheumatologist visits was also slightly higher in the community NLC group. This suggests that RPs were referring back to rheumatologist more frequently. In this model of care, rheumatologists were not providing care in the community. It is possible that isolation from medical staff in the community resulted in an increased referral for consultant opinions for matters that might have been discussed informally during a clinic visit between nurse and rheumatologist. Two factors may have contributed to this: first, the RPs did not perform intra-articular injections; if they felt one was required they referred back to the hospital clinic. Second, they were not completely independent prescribers and hence, if RA became uncontrolled and change in drug therapy was required, referral was made back to the consultant clinic. A survey of rheumatology nurses in the UK in 2009 reported that 26% of rheumatology nurses were able to independently prescribe and 33% perform intra-articular joint injections.^39^

This study compliments previous studies which have shown that NLC is an effective model when based in secondary care setting.^13–15^ ^40^ ^41^ However, it questions whether community NLC, away from medical staff, is cost-effective. The study by Ndosi provides good evidence that hospital-based NLC is cost-effective and not inferior to conventional RLC.^14^ In the present study there was no significant difference in clinical effectiveness, and no significant differences in costs There are however other reasons, such as geographical proximity of the community services to the patient’s home, to explain why particularly in rural areas community based services may develop.

While the proportion of non-biological DMARDs and opioid analgesics was relatively even and did not change during the study period, we noted a large baseline imbalance in participants using biological DMARDs, which increased over the course of the study. Although the sample sizes were small, baseline characteristics of participants who were prescribed biological DMARDS appeared similar to the rest of the study population. The reasons for this imbalance are not clear. Even when we adjust for baseline imbalance, this difference persisted; as biological drugs are very expensive, this was a major driver of the difference in costs. Use of biologics in the UK is governed by strict criteria based on disease activity. Patients recruited into the study were considered to have stable disease irrespective of treatment. It is possible that there was a selection bias away from recruiting patients taking biological agents in the hospital setting. However, analysis excluding the patients receiving biological DMARDs at baseline showed that community care may still be associated with greater costs.

### Limitations of the study

The final number of participants was less than planned; both the groups remained matched in terms of age, sex and size, but this limited our statistical power. Only 60% and 61% of participants in the community and hospital groups, respectively, completed all three sets of questionnaires. Data was only analysed for those patients for whom we had a complete set of data for the variable(s) in question, except for the sensitivity analysis in which all cases were included after multiple imputation.

The study was not randomised and there may have been disparities between the groups in characteristics that were not measured and which might have impacted on outcome. This was a pragmatic observational study and normal pattern of care was continued. Decisions about referral and appointment frequency were at the discretion of the practitioner (nursing or medical) seeing the patient. Joint counts were not performed routinely and therefore, we were unable to calculate disease activity scores. However, other measures of outcome, such as HAQ, were balanced at baseline and were assessed at 6 and 12 months.

There are limits to the generalisability of the study: in many regions, RPs are not sufficiently skilled to practice independently in out-of-hospital settings. A key limitation is prescribing ability. It is possible that some of the differences in costs and in particular, rheumatologist visits, was related to the limited prescribing authority of the nurse practitioners. Thus, in the absence of nurse prescribing, community clinics may be best run as joint clinics with rheumatologists.

## Conclusion

We conclude that community NLC may be associated with non-significant higher costs with no significant differences in clinical outcomes when compared with RLC in secondary care and this suggests a low probability that it is cost-effective. Cost-effectiveness might be improved in the community if RPs were fully able to prescribe independently and inject joints.
